# Tirzepatide and the risk of asthma exacerbations in patients with type 2 diabetes

**DOI:** 10.3389/fendo.2026.1928877

**Published:** 2026-08-25

**Authors:** Kuang-Ming Liao, Chia-Yu Kuo, Jheng-Yan Wu, Wan-Hsuan Hsu, Ya-Wen Tsai, Chih-Cheng Lai

**Affiliations:** 1 Department of Internal Medicine, Chi Mei Hospital, Chiali, Taiwan; 2 Department of Nursing, Min-Hwei Junior College of Health Care Management, Tainan, Taiwan; 3 Department of General Medicine, Chi Mei Medical Center, Tainan, Taiwan; 4 Department of Nutrition, Chi Mei Medical Center, Tainan, Taiwan; 5 Department of Public Health, College of Medicine, National Cheng Kung University, Tainan, Taiwan; 6 Division of Preventive Medicine, Chi Mei Medical Center, Tainan, Taiwan; 7 Department of Intensive Care Medicine, Chi Mei Medical Center, Tainan, Taiwan; 8 School of Medicine, College of Medicine, National Sun Yat-sen University, Kaohsiung, Taiwan

**Keywords:** asthma, asthma exacerbation, GIP receptor agonist, GLP-1 receptor agonist, tirzepatide, type 2 diabetes

## Abstract

**Background:**

Tirzepatide (TZP), a dual glucose-dependent insulinotropic polypeptide (GIP) and glucagon-like peptide-1 (GLP-1) receptor agonist, demonstrates anti-inflammatory properties that may benefit patients with asthma and type 2 diabetes (T2D). We sought to assess the comparative effectiveness of TZP versus other antidiabetic agents in reducing acute asthma exacerbations among patients with concurrent asthma and T2D.

**Methods:**

We conducted a retrospective cohort study using TriNetX US network. Adult patients with asthma and T2D initiating TZP or comparator antidiabetic agents (sulfonylureas [SU], dipeptidyl peptidase-4 inhibitors [DPP4i], sodium-glucose cotransporter 2 inhibitors [SGLT2i], or GLP-1 receptor agonists [GLP-1RA]) between January 2022 and August 2025 were included. Propensity score matching was performed for each comparison. The primary outcome was acute asthma exacerbation and secondary outcome was all-cause mortality.

**Results:**

After propensity score matching, we analyzed 25,712 patients in each TZP versus SU cohort, 23,013 in each TZP versus DPP4i cohort, 30,292 in each TZP versus SGLT2i cohort, and 19,740 in each TZP versus GLP-1RA cohort. TZP was associated with lower risk of asthma exacerbations compared with SU (HR, 0.81; 95% CI, 0.72-0.92) and DPP4i (HR, 0.82; 95% CI, 0.72-0.94). No significant differences were observed versus SGLT2i (HR, 1.12; 95% CI, 0.99-1.27; P = .064) or GLP-1RA (HR, 1.06; 95% CI, 0.91-1.23; P = .49). TZP was associated with the lower risk of all-cause mortality compared with SU, DPP4i, and SGLT2i (all P <.001).

**Conclusions:**

Among patients with coexisting asthma and T2D, TZP use was associated with a lower risk of acute asthma exacerbations compared with SU and DPP4i, and was associated with lower all-cause mortality compared with SU, DPP4i, and SGLT2i, but not compared with GLP-1RA.

## Introduction

Asthma remains a major global health burden, affecting more than 300 million individuals worldwide, with substantial regional variation in prevalence and mortality (1, 2). Recent global burden of disease analyses estimate that asthma accounts for approximately 26 million disability-adjusted life-years annually, with the greatest burden observed in low- and middle-income countries (1, 3). Asthma exacerbations represent the most severe manifestation of this chronic respiratory disease, characterized by acute worsening of symptoms that require emergency intervention (4). Thus, there remains a critical need for novel therapeutic approaches to prevent exacerbations.

Tirzepatide (TZP), a novel dual glucose-dependent insulinotropic polypeptide (GIP) and glucagon-like peptide-1 (GLP-1) receptor agonist, has demonstrated substantial efficacy in glycemic control and weight reduction among patients with type 2 diabetes (T2D) (5–7). Beyond its metabolic effects, emerging evidence suggests that tirzepatide exerts pleiotropic anti-inflammatory actions that may extend therapeutic benefits to respiratory conditions, such as asthma or obstructive sleep apnea (8–11). Prior clinical evidence also supports a protective role of GLP-1 receptor agonists (GLP-1RAs) on asthma outcomes (12). Foer et al. (12) demonstrated in a large electronic health record-based retrospective study that patients prescribed GLP-1RAs had significantly lower rates of asthma exacerbations compared with those receiving other anti-diabetic medications. While tirzepatide was not among the agents evaluated in that study, and its additional GIP receptor agonist activity may confer differential effects beyond those of GLP-1RAs alone, these findings provide meaningful clinical context supporting the plausibility of respiratory benefits with this drug class. Given the high prevalence of comorbid asthma and T2D, the potential respiratory benefits on asthma of tirzepatide warrant systematic clinical investigation.

Building on this rationale, the objective of this study was to assess the comparative effectiveness of tirzepatide versus other antidiabetic agents in reducing the risk of acute asthma exacerbations among patients with concurrent asthma and T2D. We hypothesized that TZP’s dual receptor agonism and associated anti-inflammatory properties would be associated with the lower risk of exacerbations compared with conventional diabetes therapies.

## Methods

### Data source

We conducted a retrospective cohort study utilizing data from TriNetX (13), a federated global health research network that compiles electronic health records. This platform provides access to diverse clinical variables, including diagnostic codes, procedural data, medication prescriptions, laboratory results, and genomic information. The study protocol received approval from the Institutional Review Board of Chi Mei Hospital (IRB No. 11402-E02). We conducted this study using the TriNetX US network, which contains fully de-identified patient information. Therefore, informed consent was not required, as the use of de-identified data does not necessitate an Informed Consent Form. Reporting followed the Strengthening the Reporting of Observational Studies in Epidemiology (STROBE) guidelines.

### Study design

We included adult patients (≥18 years) with a documented diagnosis of asthma and T2D, who initiated therapy with either TZP or comparator antidiabetic agents, including SU, DPP-4i, SGLT2i or GLP-1RAs, between January 1, 2022, and August 31, 2025. The study focused on new users of TZP and comparator drugs, excluding individuals with any prior or concurrent exposure to the antidiabetic medications of interest before the index date. The index date was defined as the first documented date of treatment initiation for each cohort. Patients were analyzed according to an intention-to-treat design, in which they remained in their originally assigned cohort regardless of subsequent discontinuation or switching of antidiabetic medication during follow-up. Comprehensive definitions and coding algorithms for all study variables are detailed in **Table E1**.

### Covariates and propensity score matching

Based on predefined cohort criteria, outcome definitions, and identified confounders, we constructed a covariate matrix using individual-level data from the 12-month period preceding each patient’s index date. To estimate the probability of receiving a comparator therapy, we employed logistic regression to generate propensity scores conditioned on baseline variables. Matching was performed using a one-to-one nearest-neighbor algorithm with a greedy selection strategy, applying a caliper of 0.1 times the pooled standard deviation of the logit-transformed propensity score. Patients in the smaller treatment arm were paired with the closest eligible individuals from the larger comparator group. Covariate balance post-matching was evaluated using standardized mean differences (SMDs), with values below 0.1 considered indicative of adequate balance (14).

Propensity score matching (PSM) was performed using a comprehensive set of baseline covariates to enhance comparability between treatment cohorts. Demographic variables included age, sex, race/ethnicity, and comorbidities. Clinical parameters included BMI (≥ 30 kg/m^2^) and glycemic control, defined by HbA1c (≥ 7%). T2D-related complications, encompassing renal, ophthalmic, neurological, and circulatory manifestations, were also considered. Baseline medication profiles covered lipid-lowering therapies, antihypertensive agents, glucose-lowering medications, and respiratory drugs. More specifically, the propensity score models incorporated covariates selected based on three key considerations: (1) established clinical factors known to influence both treatment selection and asthma outcomes, including age, sex, BMI, indicators of asthma severity, prior exacerbation history, and relevant comorbidities; (2) variables reflecting diabetes severity and management, such as HbA1c, the presence of diabetes-related complications, and prior use of antidiabetic medications; and (3) patterns of healthcare utilization, which may serve as proxies for disease severity, healthcare access, and overall engagement with the healthcare system. Since the TriNetX platform does not support multiple imputation, only documented values were incorporated into the propensity score model; patients lacking recorded values for specific laboratory variables were categorized based on available data. Detailed definitions and coding algorithms for all covariates are provided in **Table E2**.

### Outcomes and follow-up

The primary outcome was the incidence of acute asthma exacerbation, defined using ICD-10 diagnostic codes. Definitions and coding algorithms for all outcome measures are provided in **Table E3**. All-cause mortality was evaluated as a secondary endpoint. Follow-up commenced the day after the index date and continued until the earliest of the following events: occurrence of the specified outcome, death, the last documented clinical encounter, or one year after the index date.

### Statistical analysis

Continuous variables were reported as means ± standard deviations, and categorical variables were summarized as frequencies and percentages. To reduce confounding and improve comparability between treatment groups, PSM was applied prior to all primary and subgroup analyses. Time-to-event outcomes were assessed using Cox proportional hazards models to estimate hazard ratios (HRs) with 95% confidence intervals (CIs). Differences in event-free survival between cohorts were evaluated using Kaplan–Meier survival curves and log-rank tests.

Predefined subgroup analyses were stratified by age, sex, race, body mass index, and selected clinical characteristics, including the presence or absence of hypertension, heart failure, cardiovascular disease, chronic obstructive pulmonary disease (COPD) and the use of ICS. Additional stratifications were based on glycemic control (HbA1c ≥8% vs. <8%) and eosinophil level. For these analyses, new cohorts were created for each subgroup, and all subgroup analyses were re-conducted accordingly. To assess the robustness of findings to potential unmeasured confounding, E-values with lower confidence limit (LCL) were calculated to quantify the minimum strength of association that an unmeasured confounder would need to fully explain the observed effect estimates (15). All analyses were performed using the TriNetX analytics platform.

### Additional analysis

To address the potential for death to act as a competing event for asthma exacerbation, additional analysis was conducted using a composite outcome of all-cause mortality or acute asthma exacerbation, whichever occurred first.

## Results

### Patients’ selection

**Figure 1:**
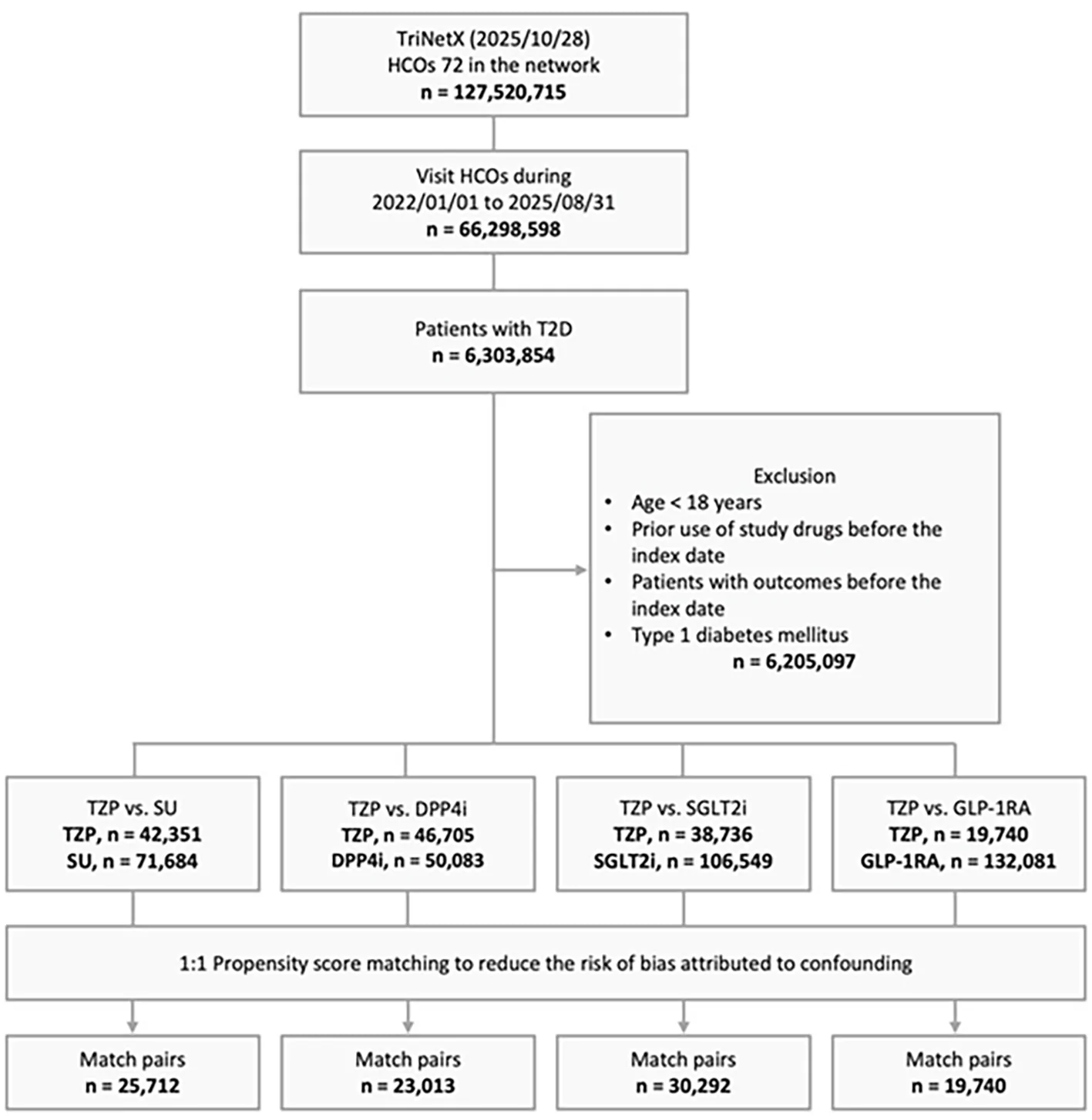
The algorithm for patient selection.

We used data from 72 healthcare organizations, encompassing a total population of 127,520,715 patients as of October 28, 2025. Among these, 66,298,598 patients had at least one healthcare encounter during the study period, and 6,303,854 of them had T2D. After applying exclusion criteria, we constructed four comparative cohorts based on exposure to TZP versus SU, DPP4i, SGLT2i, or GLP-1RA. To minimize confounding, 1:1 PSM was performed for each comparison, resulting in matched cohorts. Specifically, the TZP versus SU cohort included 25,712 patients in each group; the TZP versus DPP4i cohort included 23,013 patients in each group; the TZP versus SGLT2i cohort included 30,292 patients in each group; and the TZP versus GLP-1RA cohort included 19,740 patients in each group (**Figure 1**).

### Baseline characteristics

Before PSM, many baseline differences were noted between patients receiving TZP and those treated with SU, DPP4i, SGLT2i, or GLP-1RA (**Table E4**). Following PSM, the age, distribution of sex and race, BMI, HbA1c, comorbidities, T2D-related complications, concurrent use of cardiovascular, antihypertensive, respiratory, and glucose-lowering medications, as well as eosinophil and IgE levels, were well balanced across all four comparative cohorts (TZP versus SU, TZP versus DPP4i, TZP versus SGLT2i, and TZP versus GLP-1RA), with all SMDs below 0.1 (**Table 1**).

**Table 1:** Post-match baseline characteristics of tirzepatide and other antidiabetic medication cohorts.

Variables	TZP	SU	SMD	TZP	DPP4i	SMD	TZP	SGLT2i	SMD	TZP	GLP-1RA	SMD
Sample size in each cohort	n = 25,712			n = 23,013			n = 30,292			n = 19,740		
Age, years												
Mean (SD)	57.0 (12.6)	56.8 (13.4)	0.016	58.2 (12.3)	58.1 (13.3)	0.007	57.2 (12.5)	56.9 (13.0)	0.024	56.9 (13.1)	56.7 (13.0)	0.011
Sex, n (%)												
Female	18,177 (70.7)	18,101 (70.4)	0.007	16,086 (69.9)	16,178 (70.3)	0.007	21,719 (71.7)	21,779 (71.9)	0.003	13,896 (70.4)	13,891 (70.4)	<0.001
Male	7,536 (29.3)	7,610 (29.6)	0.007	6,903 (30.0)	6,834 (29.7)	0.007	8,572 (28.3)	8,512 (28.1)	0.004	5,843 (29.6)	5,823 (29.5)	0.001
Race, n (%)												
White	16,968 (66.0)	17,147 (66.7)	0.016	14,889 (64.7)	14,820 (64.4)	0.005	19,932 (65.8)	19,992 (66.0)	0.003	13,383 (67.8)	13,482 (68.3)	0.011
Black or African American	5,580 (21.7)	5,502 (21.4)	0.007	4,809 (20.9)	4,855 (21.1)	0.003	6,694 (22.1)	6,664 (22.0)	0.002	4,125 (20.9)	4,129 (20.9)	0.002
Asian	849 (3.3)	797 (3.1)	0.009	875 (3.8)	897 (3.9)	0.003	909 (3.0)	939 (3.1)	0.005	592 (3.0)	532 (2.7)	0.013
Other Race	771 (3.0)	745 (2.9)	0.004	782 (3.4)	805 (3.5)	0.007	879 (2.9)	910 (3.0)	0.004	552 (2.8)	531 (2.7)	0.008
Unknown Race	1,054 (4.1)	1,028 (3.9)	0.011	1,104 (4.8)	1,105 (4.8)	<0.001	1,275 (4.2)	1,241 (4.1)	0.003	731 (3.7)	710 (3.6)	0.004
Body mass index > 30 kg/m^2^, n (%)												
	17,715 (68.9)	17,869 (69.5)	0.014	14,728 (64.0)	14,889 (64.7)	0.015	21,719 (71.7)	21,961 (72.5)	0.019	14,232 (72.1)	14,272 (72.3)	0.006
Hemoglobin A1c ≥ 7%, n (%)												
	10,644 (41.1)	10,799 (42.0)	0.018	10,263 (44.6)	10,125 (44.0)	0.013	12,025 (39.7)	12,480 (41.2)	0.031	6,869 (34.8)	6,830 (34.6)	0.004
Socioeconomic, n (%)												
Potential health hazards related to socioeconomic circumstances	900 (3.5)	952 (3.7)	0.009	828 (3.6)	830 (3.6)	0.001	1,211 (4.0)	1,240 (4.1)	0.006	928 (4.7)	932 (4.7)	0.002
Comorbidities, n (%)												
Hypertension	16,690 (64.9)	16,739 (65.1)	0.004	15,027 (65.3)	14,981 (65.1)	0.004	20,053 (66.2)	20,235 (66.8)	0.013	12,752 (64.6)	12,633 (64.0)	0.012
Dyslipidemia	15,401 (59.9)	15,504 (60.3)	0.008	13,830 (60.1)	13,832 (60.1)	0.001	18,599 (61.4)	18,781 (62.0)	0.012	11,844 (60.0)	11,725 (59.4)	0.013
Ischemic heart disease	4,140 (16.1)	4,143 (16.1)	0.001	3,797 (16.5)	3,843 (16.7)	0.006	4,725 (15.6)	4,695 (15.5)	0.002	3,099 (15.7)	3,059 (15.5)	0.010
Neoplasms	4,937 (19.2)	4,910 (19.1)	0.005	4,464 (19.4)	4,442 (19.3)	0.003	6,088 (20.1)	6,149 (20.3)	0.005	4,046 (20.5)	3,967 (20.1)	0.011
Chronic kidney disease	3,034 (11.8)	3,086 (12.0)	0.006	3,129 (13.6)	3,152 (13.7)	0.002	3,665 (12.1)	3,877 (12.8)	0.021	2,368 (12.0)	2,289 (11.6)	0.011
Overweight and obesity	12,035 (46.8)	12,240 (47.6)	0.016	9,872 (42.9)	9,941 (43.2)	0.008	15,236 (50.3)	15,630 (51.6)	0.026	10,205 (51.7)	10,245 (51.9)	0.005
Obstructive sleep apnea	6,635 (25.8)	6,764 (26.3)	0.009	5,500 (23.9)	5,569 (24.2)	0.005	8,633 (28.5)	8,754 (28.9)	0.008	4,046 (20.5)	3,967 (20.1)	0.009
Heart failure	2,829 (11.0)	2,831 (11.0)	<0.001	2,601 (11.3)	2,646 (11.5)	0.006	3,120 (10.3)	3,333 (11.0)	0.023	2,309 (11.7)	2,250 (11.4)	0.010
Atrial fibrillation and flutter	1,928 (7.5)	1,931 (7.5)	0.001	1,795 (7.8)	1,796 (7.8)	0.001	2,180 (7.2)	2,302 (7.6)	0.013	1,559 (7.9)	1,519 (7.7)	0.007
Allergic rhinitis	1,646 (6.4)	1,648 (6.4)	0.003	1,472 (6.4)	1,475 (6.4)	0.002	1,939 (6.4)	2,000 (6.6)	0.007	1,342 (6.8)	1,283 (6.5)	0.010
Urticaria	257 (1.0)	259 (1.0)	0.007	230 (1.0)	234 (1.0)	0.003	333 (1.1)	337 (1.1)	0.001	236 (1.2)	241 (1.2)	0.006
Food allergy status	283 (1.1)	284 (1.1)	0.006	207 (0.9)	234 (1.0)	0.005	331 (1.1)	334 (1.1)	0.001	217 (1.1)	220 (1.1)	0.004
Chronic allergic conjunctivitis	81 (0.3)	79 (0.3)	0.013	69 (0.3)	71 (0.3)	0.002	90 (0.3)	93 (0.3)	0.011	79 (0.4)	59 (0.3)	0.004
Gastro-esophageal reflux disease	7,970 (31.0)	8,022 (31.2)	0.003	7,157 (31.1)	7,249 (31.5)	0.008	9,723 (32.1)	9,784 (32.3)	0.005	6,336 (32.1)	6,178 (31.3)	0.017
Depressive episode	4,886 (19.0)	4,936 (19.2)	0.006	4,326 (18.8)	4,372 (19.0)	0.006	5,785 (19.1)	5,877 (19.4)	0.008	3,770 (19.1)	3,768 (19.1)	0.001
Nicotine dependence	2,674 (10.4)	2,675 (10.4)	<0.001	2,324 (10.1)	2,439 (10.6)	0.017	2,999 (9.9)	3,120 (10.3)	0.012	1,954 (9.9)	1,974 (10.0)	0.003
Alcohol related disorders	462 (1.8)	465 (1.8)	0.005	391 (1.7)	414 (1.8)	0.008	515 (1.7)	516 (1.7)	0.004	395 (2.0)	399 (2.0)	0.002
Hypothyroidism	4,090 (15.9)	4,062 (15.8)	0.003	3,660 (15.9)	3,664 (15.9)	0.003	4,968 (16.4)	5,000 (16.5)	0.004	3,336 (16.9)	3,257 (16.5)	0.011
Hyperthyroidism	283 (1.1)	308 (1.2)	0.001	276 (1.2)	299 (1.3)	0.006	393 (1.3)	395 (1.3)	0.002	236 (1.2)	256 (1.3)	0.002
Type 2 diabetes complications												
Kidney complications	2,802 (10.9)	2,905 (11.3)	0.012	2,807 (12.2)	2,876 (12.5)	0.008	3,362 (11.1)	3,574 (11.8)	0.023	2,033 (10.3)	1,993 (10.1)	0.006
Neurological complications	3,162 (12.3)	3,188 (12.4)	0.002	2,945 (12.8)	3,014 (13.1)	0.010	3,786 (12.5)	3,937 (13.0)	0.015	2,052 (10.4)	2,013 (10.2)	0.004
Circulation complications	1,439 (5.6)	1,441 (5.6)	<0.001	1,242 (5.4)	1,288 (5.6)	0.007	1,817 (6.0)	1,878 (6.2)	0.010	1,125 (5.7)	1,127 (5.7)	0.001
Ophthalmic complications	978 (3.8)	1,005 (3.9)	0.002	943 (4.1)	990 (4.3)	0.009	1,211 (4.0)	1,302 (4.3)	0.011	631 (3.2)	635 (3.2)	0.002
Medication, n (%)												
Lipid modifying agents	11,236 (43.7)	11,210 (43.6)	0.005	10,516 (45.7)	10,470 (45.5)	0.003	12,904 (42.6)	12,964 (42.8)	0.012	8,409 (42.6)	8,290 (42.0)	0.014
Antihypertensives	2,391 (9.3)	2,365 (9.2)	0.002	2,025 (8.8)	1,956 (8.5)	0.009	2,726 (9.0)	2,817 (9.3)	0.011	1,816 (9.2)	1,835 (9.3)	0.002
Inhaled corticosteroid	10,104 (39.3)	10,027 (39.0)	0.007	9,159 (39.8)	9,090 (39.5)	0.006	12,540 (41.4)	12,510 (41.3)	0.003	8,113 (41.1)	8,014 (40.6)	0.010
Systemic corticosteroid	5,091 (19.8)	5,095 (19.8)	0.002	4,304 (18.7)	4,211 (18.3)	0.010	5,906 (19.5)	5,909 (19.5)	0.001	4,086 (20.7)	4,087 (20.7)	<0.001
Inhaled beta-2-adrenoreceptor agonists	10,106 (39.3)	10,028 (39.0)	0.007	10,655 (46.3)	10,585 (46.0)	0.006	14,328 (47.3)	14,297 (47.2)	0.002	9,258 (46.9)	9,259 (46.9)	<0.001
Indacaterol	0 (0)	10 (0.0)	0.027	0 (0)	11 (0.0)	0.029	0 (0)	15 (0.0)	0.026	0 (0)	0 (0)	–
Olodaterol	128 (0.5)	133 (0.5)	0.003	138 (0.6)	115 (0.5)	0.009	151 (0.5)	181 (0.6)	0.004	118 (0.6)	121 (0.6)	0.001
Formoterol	2,519 (9.8)	2,496 (9.7)	0.001	2,305 (10.0)	2,326 (10.1)	0.002	3,089 (10.2)	3,180 (10.5)	0.009	2,092 (10.6)	2,096 (10.6)	0.001
Salmeterol	2,082 (8.1)	2,056 (8.0)	0.005	1,841 (8.0)	1,795 (7.8)	0.006	2,302 (7.6)	2,306 (7.6)	0.001	1,519 (7.7)	1,500 (7.6)	0.004
Leukotriene receptor antagonists	3,240 (12.6)	3,265 (12.7)	0.003	2,922 (12.7)	2,899 (12.6)	0.002	4,150 (13.7)	4,240 (14.0)	0.008	2,546(12.9)	2,526 (12.8)	0.003
Tiotropium	720 (2.8)	723 (2.8)	0.003	690 (3.0)	692 (3.0)	0.002	848 (2.8)	851 (2.8)	0.005	513 (2.6)	493 (2.5)	0.006
Umeclidinium	823 (3.2)	797 (3.1)	0.008	782 (3.4)	760 (3.3)	0.007	1,211 (4.0)	1,241 (4.1)	0.006	848 (4.3)	868 (4.4)	0.005
Xanthines	180 (0.7)	154 (0.6)	0.011	184 (0.8)	187 (0.8)	0.004	181 (0.6)	212 (0.7)	0.011	99 (0.5)	118 (0.6)	0.008
Proton pump inhibitors	7,251 (28.2)	7,174 (27.9)	0.008	6,903 (30.0)	7,134 (30.1)	0.001	8,905 (29.4)	8,966 (29.6)	0.003	5,625 (28.5)	5,448 (27.6)	0.020
Biguanides	9,256 (36.0)	9,335 (36.3)	0.005	8,790 (38.2)	8,675 (37.7)	0.009	10,735 (35.5)	10,905 (36.0)	0.010	6,494 (32.9)	6,533 (33.1)	0.005
Thiazolidinediones	412 (1.6)	413 (1.6)	<0.001	483 (2.1)	506 (2.2)	0.007	514 (1.7)	520 (1.7)	0.002	315 (1.6)	355 (1.8)	0.011
Alpha glucosidase inhibitors	26 (0.1)	26 (0.1)	<0.001	46 (0.2)	48 (0.2)	0.001	30 (0.1)	34 (0.1)	0.003	19 (0.1)	38 (0.2)	0.001
Insulins and analogues	6,299 (24.5)	6,402 (24.9)	0.010	6,006 (26.1)	6,075 (26.4)	0.007	7,482 (24.7)	7,603 (25.1)	0.026	3,790 (19.2)	3,791 (19.2)	<0.001
Sulfonylureas	–	–	–	2,462 (10.7)	2,485 (10.8)	0.003	2,605 (8.6)	2,665 (8.8)	0.008	1,500 (7.6)	1,559 (7.9)	0.012
Dipeptidyl peptidase 4 inhibitors	1,132 (4.4)	1,234 (4.8)	0.018	–	–	–	1,151 (3.8)	1,272 (4.2)	0.020	947 (4.8)	963 (4.9)	0.002
Sodium-glucose co-transporter 2 inhibitors	3,368 (13.1)	3,366 (13.1)	0.002	3,175 (13.8)	3,129 (13.6)	0.006	–	–	–	2,605 (13.2)	2,585 (13.1)	0.003
Glucagon-like peptide-1 analogues	5,296 (20.6)	5,298 (20.6)	0.001	3,820 (16.6)	3,659 (15.9)	0.018	9,935 (32.8)	10,451 (34.5)	0.038	–	–	–
Lab data												
Eosinophils in blood, n (%)*	19,387 (75.4)	19,745 (76.8)		17,281 (75.1)	17,353 (75.4)		23,505 (77.6)	23,810 (78.6)		15,928 (80.7)	15,933 (80.7)	
≤ 150/uL	7,354 (28.6)	7,713 (30.0)	0.008	6,696 (29.1)	6,697 (29.1)	<0.001	9,057 (29.9)	9,148 (30.2)	0.007	6,079 (30.8)	6,139 (31.1)	0.005
150-300/uL	6,865 (26.7)	6,967 (27.1)	0.009	6,029 (26.2)	6,098 (26.5)	0.007	8,360 (27.6)	8,483 (28.0)	0.009	5,704 (28.9)	5,645 (28.6)	0.008
≥ 300/uL	5,168 (20.1)	5,065 (19.7)	0.008	4,556 (19.8)	4,558 (19.8)	<0.001	6,088 (20.1)	6,179 (20.4)	0.007	4,145 (21.0)	4,149 (21.0)	0.002
IgE in blood, n (%)*	298 (1.2)	247 (1.0)		265 (1.2)	241 (1.1)		358 (1.2)	363 (1.2)		297 (1.5)	290 (1.5)	
≥ 100 IU/ml	129 (0.5)	102 (0.4)	0.006	115 (0.5)	113 (0.5)	0.005	151 (0.5)	152 (0.5)	0.001	98 (0.5)	118 (0.6)	0.009

TZP, Tirzepatide; SU, sulfonylurea; GLP-1RA, glucagon-like peptide-1 receptor agonist; DPP4i, dipeptidyl peptidase 4 inhibitor; SGLT2i, sodium-glucose cotransporter 2 inhibitor; SMD, standardized mean difference.

*Number of patients with available data.

### TZP versus SU cohort

**Figure 2:**
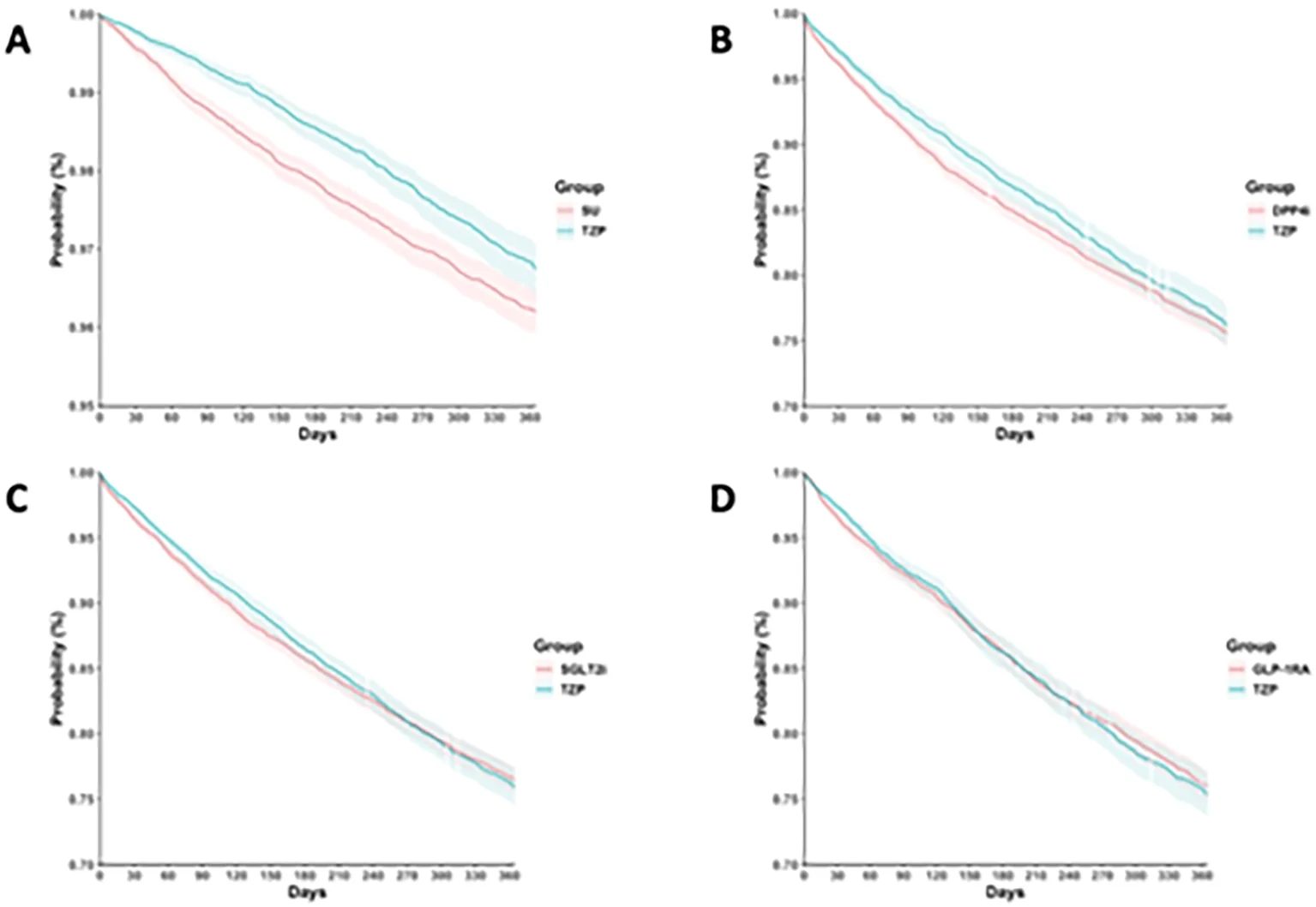
Kaplan-Meier curves showing time to event-free survival for the primary outcome for tirzepatide (TZP) compared with sulfonylureas (SU) **(A)**, DPP-4 inhibitors (DPP-4i) **(B)**, SGLT2 inhibitors (SGLT2i) **(C)**, and GLP-1 receptor agonists (GLP-1RA) **(D)**.

Compared with the SU group, the TZP group was associated with a lower incidence of asthma acute exacerbation (18.4 vs 26.2 events per 1000 person-years; HR, 0.81; 95% CI, 0.72–0.92; P = .0012; P for proportionality > 0.05, **Table 2**). The corresponding E-value was 1.77 (95% LCL, 1.39). Kaplan–Meier analysis showed a lower cumulative probability of asthma acute exacerbation in the TZP group than in the SU group (log-rank P = 0.0012; **Figure 2A**). Stratified analyses demonstrated consistent associations, with HRs below 1 across all evaluated subgroups (**Figure E1**).

**Table 2:** Hazard ratio of primary and secondary outcomes between tirzepatide and other comparator groups.

Comparator	Events (n)	Incidence rate per 1000 PYs	Events (n)	Incidence rate per 1000 PYs	HR (95% CI)	*P* value	E-value (95% LCL)
Primary outcome: asthma acute exacerbation							
Tirzepatide (n = 25,712) vs. SU (n = 25,712)	393	18.4	674	26.2	0.81 (0.72,0.92)	0.0012	1.77 (1.39)
Tirzepatide (n = 23,013) vs. DPP4i (n = 23,013)	339	17.9	592	25.7	0.82 (0.72,0.94)	0.0045	1.74 (1.32)
Tirzepatide (n = 30,292) vs. SGLT2i (n = 30,292)	486	18.7	570	18.8	1.12 (0.99,1.27)	0.0643	1.49 (1.00)
Tirzepatide (n = 19,740) vs. GLP-1RA (n = 19,740)	307	19.1	387	19.6	1.06 (0.91,1.23)	0.4878	1.31 (1.00)
Secondary outcomes: all-cause mortality							
Tirzepatide (n = 25,712) vs. SU (n = 25,712)	99	4.6	305	11.9	0.42 (0.34,0.53)	<0.0001	4.19 (3.18)
Tirzepatide (n = 23,013) vs. DPP4i (n = 23,013)	105	5.5	302	13.1	0.47 (0.37,0.58)	<0.0001	3.68 (2.84)
Tirzepatide (n = 30,292) vs. SGLT2i (n = 30,292)	124	4.8	274	9.1	0.57 (0.46,0.70)	<0.0001	2.90 (2.21)
Tirzepatide (n = 19,740) vs. GLP-1RA (n = 19,740)	92	5.7	134	6.8	0.90 (0.72,1.85)	0.4524	1.46 (1.00)

CI, confidence interval; DPP4i, dipeptidyl peptidase 4 inhibitor; GLP-1RA, glucagon-like peptide-1 receptor agonist; HR, hazard ratio; LCL, lower confidence limit; SU, sulfonylurea; SGLT2i, sodium-glucose cotransporter 2 inhibitor.

For secondary outcomes, the TZP group was associated with a lower risk of all-cause mortality compared with the SU group (HR, 0.42; 95% CI, 0.34–0.53; P <.0001; E-value, 4.19 [95% LCL, 3.18]).

### TZP versus DPP4i cohort

When compared with the DPP4i group, TZP use was associated with fewer asthma acute exacerbations (17.9 vs 25.7 events per 1000 person-years; HR, 0.82; 95% CI, 0.72–0.94; P = .0045; P for proportionality > 0.05, **Table 2**). The E-value for this association was 1.74 (95% LCL, 1.32). Kaplan–Meier curves illustrated a lower cumulative incidence of asthma acute exacerbation among TZP users (log-rank P = 0.0045; **Figure 2B**). Subgroup analyses revealed directionally similar associations across most strata, with HRs below 1 in each category (**Figure E2**).

In terms of secondary outcomes, patients receiving TZP was associated with a lower risk of all-cause mortality than those treated with DPP4i (HR, 0.47; 95% CI, 0.37–0.58; P <.0001; E-value, 3.68 [95% LCL, 2.84]).

### TZP versus SGLT2i cohort

There was no significant difference in the incidence of asthma acute exacerbation between the TZP and SGLT2i groups (18.7 vs 18.8 events per 1000 person-years; HR, 1.12; 95% CI, 0.99–1.27; P = .064; P for proportionality > 0.05, **Table 2**). Kaplan–Meier analysis demonstrated comparable cumulative risks of asthma acute exacerbation between the two groups (log-rank P = 0.0643; **Figure 2C**). Subgroup analyses revealed broadly consistent findings, showing no significant differences across most strata (**Figure E3**). Nevertheless, TZP was associated with a higher risk of asthma acute exacerbation among older adults (≥65 years), patients with cardiovascular disease or heart failure, and those with eosinophil counts of 150–300/uL.

For secondary outcomes, TZP was associated with a significantly lower risk of all-cause mortality than SGLT2i (HR, 0.57; 95% CI, 0.46–0.70; P = .002; E-value, 2.90 [95% LCL, 2.21]).

### TZP versus GLP-1RA cohort

The incidence of asthma acute exacerbation was similar between the TZP and GLP-1RA groups (19.1 vs 19.6 events per 1000 person-years; HR, 1.06; 95% CI, 0.91–1.23; P = .49; P for proportionality > 0.05, **Table 2**). The Kaplan–Meier analysis showed similar cumulative incidence curves between groups (log-rank P = 0.4878; **Figure 2D**). Across all prespecified subgroups, the results were consistent (**Figure E4**). No significant difference in all-cause mortality was observed between the two groups (HR, 0.90; 95% CI, 0.72–1.85; P = .45; E-value, 1.46 [95% LCL, 1.00]).

### Additional analysis

In the sensitivity analysis using a composite outcome of acute asthma exacerbation or all-cause mortality, tirzepatide was associated with a lower risk compared with SU (HR, 0.70; 95% CI, 0.63–0.77), DPP-4i (HR, 0.62; 95% CI, 0.55–0.70), and SGLT2i (HR, 0.84; 95% CI, 0.76–0.93). No significant difference was observed between tirzepatide and GLP-1RA (HR, 1.02; 95% CI, 0.90–1.15) (**Supplementary Table 5**). Most findings were consistent with the primary Cox model results.

## Discussion

In this large, real-world comparative effectiveness study of patients with coexisting asthma and T2D, TZP use was associated with a lower risk of acute asthma exacerbations compared with SU and DPP4i. These findings were consistent across multiple subgroups and supported by Kaplan–Meier analyses. In contrast, no significant differences in exacerbation risk were observed when TZP was compared with SGLT2i or GLP-1RAs, suggesting that these therapies may have potential anti-inflammatory effects and offer similar benefits in reducing asthma exacerbations. Notably, certain subgroups, such as older adults and those with cardiovascular disease, exhibited higher exacerbation risk with tirzepatide versus SGLT2is, warranting further investigation into differential responses across phenotypes.

Our findings differ from those reported by Foer et al. (12), who observed lower exacerbation rates with GLP-1RA compared with SGLT2i over a 6-month follow-up. Several methodological and clinical differences likely explain this discrepancy. First, study populations differed in baseline characteristics, including asthma severity, comorbidities, and cardiometabolic risk profiles, which may influence both treatment selection and outcomes. Second, differences in follow-up duration may capture distinct patterns of exacerbation risk. Third, variations in outcome definitions and ascertainment further limit direct comparability. Collectively, these factors suggest that the studies provide complementary, rather than conflicting, evidence across different clinical contexts.

In addition to respiratory outcomes, TZP use was associated with a lower risk of all-cause mortality compared with SU, DPP4i, and SGLT2i, although not compared with GLP-1RA. However, given the observational design, residual confounding and treatment selection, such as clinicians preferentially prescribing TZP to patients perceived as healthier or better able to tolerate the medication, cannot be excluded and may have contributed to these findings. The larger magnitude of association observed for SU and DPP4i compared with SGLT2i and GLP-1RA is consistent with prior evidence on the cardiometabolic profile of tirzepatide, but should not be interpreted as evidence of a causal survival benefit. Accordingly, the mortality findings should be considered exploratory and hypothesis-generating rather than confirmatory, and they warrant further evaluation in prospective studies before conclusions can be drawn regarding a survival benefit of TZP in this population.

The observed respiratory and survival associations with TZP may be partly explained by its dual GIP/GLP-1R agonism. GLP-1R activation improves glycemic control, promotes weight loss, and reduces cardiometabolic risk, and GLP-1RAs more broadly exert anti-inflammatory and immunomodulatory effects across chronic inflammatory conditions, including obesity, cardiovascular disease, and asthma (9, 16–20). GLP-1RAs may also directly modulate airway inflammation through effects on eosinophils, which express GLP-1 receptors; GLP-1 analogs have been shown to reduce eosinophil activation markers and suppress IL-4, IL-8, and IL-13, though not IL-5 (21–24). However, because TZP produces substantial weight loss, part of the observed reduction in exacerbations may be mediated through improvements in obesity-related airway mechanics and systemic inflammation rather than through a direct pharmacologic anti-inflammatory effect. Weight reduction itself may therefore partially mediate the observed respiratory benefit, and the present study cannot distinguish weight-loss-mediated effects from direct receptor-mediated pharmacologic mechanisms. These broader metabolic and anti-inflammatory effects, whether weight-loss-mediated or direct, may collectively contribute to the respiratory and survival associations observed with TZP.

The absence of a significant difference in asthma exacerbation risk between tirzepatide and GLP-1RAs may suggest that the additional GIP receptor agonist activity of tirzepatide does not have a measurable incremental benefit for asthma outcomes beyond that achieved through GLP-1 receptor activation alone. However, this interpretation should be approached cautiously. Our study was not specifically designed to isolate the independent contribution of GIP receptor signaling or to compare the mechanistic effects of individual incretin-based therapies. In addition, residual confounding, differences in prescribing patterns, and the relatively low event rate may have limited our ability to detect small but clinically meaningful differences between treatment groups. Furthermore, the role of GIP signaling in airway inflammation and asthma pathophysiology remains incompletely understood.

This study has several strengths. First, it used a large, multicenter real-world dataset encompassing diverse patient populations across the United States, enhancing generalizability. Second, the use of rigorous PSM across four comparator cohorts minimized confounding and ensured balanced baseline characteristics. Third, the consistency of findings across multiple stratified analyses lends robustness to the observed associations. Finally, the inclusion of both respiratory and mortality outcomes provides a comprehensive assessment of TZP’s potential dual benefit in patients with coexisting asthma and T2D.

This study has several limitations. First, as an observational analysis, residual confounding cannot be fully excluded despite propensity score matching and E-value estimation. In particular, important clinical factors, including asthma severity, pulmonary function, inhaled corticosteroid dose, biologic therapy use, medication adherence, and obesity severity, were not captured within the TriNetX platform and could not be incorporated into the propensity score model; residual confounding from these unmeasured factors should be considered when interpreting our findings. Second, medication selection may also be influenced by unmeasured socioeconomic factors. Although an ICD-10-CM socioeconomic hazard code was included in the matching model, it was recorded in only 3.5-4.7% of participants, limiting its ability to adjust for socioeconomic status, insurance type, or health literacy. Third, several data-completeness limitations remain. Fourth, our findings are directly applicable to adults with coexisting COPD and T2D who received care within U.S. healthcare organizations contributing to the TriNetX network, and caution should be exercised in extrapolating these results to other healthcare systems, populations, or countries with differing prescribing patterns and care delivery models. Fifth, missing data were observed for several key covariates across the matched cohorts, with proportions ranging from approximately 27.7% to 36.0% for BMI, 55.4% to 65.4% for HbA1c, 19.3% to 24.9% for eosinophil count, and 98.5% to 99.0% for IgE (**Supplementary Table 6**). Multiple imputation was not feasible given the federated, non-exportable structure of the TriNetX platform, and these variables were therefore analyzed as complete cases. Sixth, the low observed prevalence of allergic rhinitis (6.5%) likely reflects under-coding rather than true absence, limiting our ability to adjust for this comorbidity. Seventh, patients with prior asthma exacerbations were excluded at baseline, resulting in a relatively low event rate (18–26 per 1,000 person-years); our findings are therefore most applicable to patients with relatively stable asthma and may not generalize to those with more severe or unstable disease. Eighth, a formal competing-risks analysis could not be performed, as Fine-Gray modeling requires patient-level data unavailable through TriNetX; a supplementary composite-outcome analysis (mortality or exacerbation, whichever occurred first) was used to partially address this, though residual bias cannot be fully excluded. Finally, given the large number of subgroup analyses performed, the possibility of false-positive findings due to multiple comparisons cannot be excluded, and these subgroup results should be interpreted as exploratory and hypothesis-generating.

## Conclusions

Among patients with coexisting asthma and T2D, TZP use was associated with a lower risk of acute asthma exacerbations compared with SU and DPP4i, and was associated with lower all-cause mortality compared with SU, DPP4i, and SGLT2i, but not compared with GLP-1RA. Further prospective studies are warranted to validate these observations and elucidate underlying mechanisms.

## Data Availability

The original contributions presented in the study are included in the article/**Supplementary Material**. Further inquiries can be directed to the corresponding author.
