# Supplementary material for: Tirzepatide and the risk of asthma exacerbations in patients with type 2 diabetes
Source: Front Endocrinol (Lausanne). 2026 Aug 25;17:1928877. doi: 10.3389/fendo.2026.1928877 (PMC13549827; doi:10.3389/fendo.2026.1928877)
Supplement: Supplementary file 1 [file DataSheet1.docx]

**Supplemental Table 1.** Demographic, diagnostic, procedural, medication, visit, and laboratory codes utilized in the definition of the cohorts.

| **Category** | **Code** | **Description** |
| --- | --- | --- |
| **T2D** | | |
| Diagnosis | UMLS:ICD10CM:E11 | Type 2 diabetes mellitus |
| **Anti-diabetic medication** | | |
| Medication | NLM:RXNORM:2601723 | Tirzepatide |
| Medication | NLM:ATC:A10BJ | Glucagon-like peptide-1 (GLP-1) analogues |
| Medication | NLM:ATC:A10BH | Dipeptidyl peptidase 4 (DPP-4) inhibitors |
| Medication | NLM:ATC:A10BK | Sodium-glucose co-transporter 2 (SGLT2) inhibitors |
| Medication | NLM:ATC:A10BB | Sulfonylurea |

**Supplemental Table 2.** Definitions of covariates coding used in this study.

| **Code** | **Description** |
| --- | --- |
| AI | Age at Index |
| F | Female |
| M | Male |
| Mar-06 | White |
| May-54 | Black or African American |
| Sep-28 | Asian |
| Jan-31 | Other Race |
| UNK | Unknown Race |
| 9083 | Body mass index, at least 30 kg/m2 |
| 9037 | Hemoglobin A1c/Hemoglobin.total in Blood, at least 7% |
| I10 | Hypertension |
| E78 | Dyslipidemia |
| J40-J4A | Chronic lower respiratory diseases |
| I20-I25 | Ischemic heart disease |
| I60-I69 | Cerebrovascular diseases |
| C00-D49 | Neoplasms |
| N18 | Chronic kidney disease |
| E66 | Overweight and obesity |
| G47.33 | Obstructive sleep apnea |
| I50 | Heart failure |
| I48 | Atrial fibrillation and flutter |
| J30.9 | Allergic rhinitis |
| L50 | Urticaria |
| Z91.01 | Food allergy status |
| H10.45 | Chronic allergic conjunctivitis |
| K21 | Gastro-esophageal reflux disease |
| F32 | Depressive episode |
| F17 | Nicotine dependence |
| F10 | Alcohol related disorders |
| E03 | Hypothyroidism |
| E05 | Hyperthyroidism |
| E11.2 | Type 2 diabetes with kidney complications |
| E11.4 | Type 2 diabetes with neurological complications |
| E11.5 | Type 2 diabetes with circulation complications |
| E11.3 | Type 2 diabetes with ophthalmic complications |
| C10 | Lipid modifying agents |
| Z55-Z65 | Potential health hazards related to socioeconomic circumstances |
| C02 | Antihypertensives |
| R03BA | Inhaled corticosteroid |
| 8640 | Systemic corticosteroid |
| M01A | Anti-inflammatory products, non-steroids |
| R03AC | Inhaled beta-2-adrenoreceptor agonists |
| R03DC | Leukotriene receptor antagonists |
| 69120 | Tiotropium |
| 1487514 | Umeclidinium |
| R03DX | Other systemic drugs for obstructive airway diseases |
| R03DA | Xanthines |
| A02BC | Proton pump inhibitors |
| A10BA | Biguanides |
| A10BG | Thiazolidinediones |
| A10BF | Alpha glucosidase inhibitors |
| A10A | Insulins and analogues |
| A10BB | Sulfonylureas |
| A10BH | Dipeptidyl peptidase 4 inhibitors |
| A10BK | Sodium-glucose co-transporter 2 inhibitors |
| A10BJ | Glucagon-like peptide-1 analogues |
| LG32849-8 | Eosinophils [#/volume] in Blood ≤ 150/uL |
| LG32849-8 | Eosinophils [#/volume] in Blood 150-300/uL |
| LG32849-8 | Eosinophils [#/volume] in Blood ≥ 300/uL |
| LG5901-6 | IgE [Units/volume] in Serum, Plasma or Blood ≥ 100 IU/ml |

**Supplemental Table 3.** Definitions of outcomes coding used in this study.

| **Code** | **Description** |
| --- | --- |
| **Asthma acute exacerbation** | |
| J45.21 | Mild intermittent asthma with (acute) exacerbation |
| J45.31 | Mild persistent asthma with (acute) exacerbation |
| J45.41 | Moderate persistent asthma with (acute) exacerbation |
| J45.51 | Severe persistent asthma with (acute) exacerbation |
| J45.901 | Unspecified asthma with (acute) exacerbation |
| **Mortality** | |
| Deceased | Deceased |
| R99 | Ill-defined and unknown cause of mortality |

**Supplemental Table 4**. Pre-match baseline characteristics of TZP and other antidiabetic medication cohorts.

| Variables | TZP | SU | SMD | TZP | DPP4i | SMD | TZP | SGLT2i | SMD | TZP | GLP-1RA | SMD |
| --- | --- | --- | --- | --- | --- | --- | --- | --- | --- | --- | --- | --- |
| Sample size in each cohort | n = 42,351 | n = 71,684 |  | n = 46,705 | n = 50,083 |  | n = 38,736 | n = 106,549 |  | n = 19,740 | n = 132,081 |  |
| Age, years |  |  |  |  |  |  |  |  |  |  |  |  |
| Mean (SD) | 55.8 (12.7) | 59.3 (13.4) | 0.271 | 56.0 (12.7) | 61.1 (13.3) | 0.396 | 55.5 (12.8) | 62.0 (13.1) | 0.503 | 56.9 (13.1) | 56.7 (13.1) | 0.019 |
| Sex, n (%) |  |  |  |  |  |  |  |  |  |  |  |  |
| Female | 31,128 (73.5) | 47,096 (65.7) | 0.172 | 34,048 (72.9) | 34,006 (67.9) | 0.109 | 29,246 (75.5) | 64,888 (60.9) | 0.318 | 13,896 (70.4) | 92,720 (70.2) | 0.004 |
| Male | 11,223 (26.5) | 24,587 (34.3) | 0.172 | 12,657 (27.1) | 16,076 (32.1) | 0.109 | 9,490 (24.5) | 41,661 (39.1) | 0.317 | 5,843 (29.6) | 39,228 (29.7) | 0.003 |
| Race, n (%) |  |  |  |  |  |  |  |  |  |  |  |  |
| White | 28,248 (66.7) | 44,515 (62.1) | 0.097 | 31,152 (66.7) | 28,998 (57.9) | 0.182 | 25,992 (67.1) | 64,142 (60.2) | 0.143 | 13,383 (67.8) | 80,701 (61.1) | 0.140 |
| Black or African American | 9,190 (21.7) | 16,487 (23.0) | 0.030 | 9,761 (20.9) | 11,068 (22.1) | 0.029 | 8,444 (21.8) | 25,572 (24.0) | 0.050 | 4,125 (20.9) | 32,227 (24.4) | 0.083 |
| Asian | 1,228 (2.9) | 3,297 (4.6) | 0.089 | 1,401 (3.0) | 3,505 (7.0) | 0.184 | 1,007 (2.6) | 5,434 (5.1) | 0.132 | 594 (3.0) | 4,886 (3.7) | 0.042 |
| Other Race | 1,226 (2.9) | 2,365 (3.3) | 0.021 | 1,541 (3.3) | 1,903 (3.8) | 0.030 | 1,085 (2.8) | 4,516 (3.3) | 0.026 | 552 (2.8) | 4,622 (3.5) | 0.041 |
| Unknown Race | 1,653 (3.9) | 3,225 (4.5) | 0.031 | 1,915 (4.1) | 3,155 (6.3) | 0.096 | 1,472 (3.8) | 5,327 (5.0) | 0.057 | 734 (3.7) | 6,471 (4.9) | 0.061 |
| Body mass index > 30 kg/m², n (%) |  |  |  |  |  |  |  |  |  |  |  |  |
|  | 32,298 (76.5) | 41,003 (57.2) | 0.418 | 34,702 (74.3) | 27,295 (54.5) | 0.422 | 29,711 (76.7) | 64,356 (60.4) | 0.358 | 14,232 (72.1) | 91,005 (68.9) | 0.069 |
| Hemoglobin A1c ≥ 7%, n (%) |  |  |  |  |  |  |  |  |  |  |  |  |
|  | 13,806 (32.6) | 38,852 (54.2) | 0.446 | 16,487 (35.3) | 27,395 (54.7) | 0.398 | 12,279 (31.7) | 54,660 (51.3) | 0.406 | 6,869 (34.8) | 65,776 (49.8) | 0.307 |
| Comorbidities, n (%) |  |  |  |  |  |  |  |  |  |  |  |  |
| Hypertension | 27,740 (65.5) | 48,816 (68.1) | 0.054 | 30,592 (65.5) | 34,757 (69.4) | 0.083 | 25,062 (64.7) | 77,248 (72.5) | 0.168 | 12,752 (64.6) | 88,362 (66.9) | 0.050 |
| Dyslipidemia | 26,257 (62.0) | 43,512 (60.7) | 0.026 | 28,677 (61.4) | 31,902 (63.7) | 0.049 | 23,319 (60.2) | 72,986 (68.5) | 0.175 | 11,844 (60.0) | 80,305 (60.8) | 0.017 |
| Ischemic heart disease | 6,564 (15.5) | 12,759 (17.8) | 0.063 | 7,239 (15.5) | 9,966 (19.9) | 0.114 | 5,036 (13.0) | 32,711 (30.7) | 0.439 | 3,099 (15.7) | 21,925 (16.6) | 0.024 |
| Neoplasms | 9,148 (21.6) | 12,329 (17.2) | 0.111 | 9,901 (21.2) | 9,565 (19.1) | 0.052 | 8,173 (21.1) | 21,843 (20.5) | 0.016 | 4,046 (20.5) | 24,434 (18.5) | 0.052 |
| Chronic kidney disease | 4,913 (11.6) | 8,888 (12.4) | 0.025 | 5,605 (12.0) | 8,413 (16.8) | 0.138 | 3,912 (10.1) | 24,719 (23.2) | 0.356 | 2,368 (12.0) | 17,038 (12.9) | 0.030 |
| Overweight and obesity | 24,945 (58.9) | 24,946 (34.8) | 0.497 | 26,762 (57.3) | 16,727 (33.4) | 0.495 | 22,661 (58.5) | 43,685 (41.0) | 0.357 | 10,205 (51.7) | 62,606 (47.4) | 0.084 |
| Obstructive sleep apnea | 13,891 (32.8) | 13,118 (18.3) | 0.339 | 14,946 (32.0) | 9,014 (18.0) | 0.327 | 12,163 (31.4) | 27,809 (26.1) | 0.117 | 6,020 (30.5) | 34,341 (26.0) | 0.101 |
| Heart failure | 4,786 (11.3) | 8,315 (11.6) | 0.008 | 5,324 (11.4) | 6,460 (12.9) | 0.044 | 3,021 (7.8) | 31,219 (29.3) | 0.575 | 2,309 (11.7) | 15,453 (11.7) | 0.001 |
| Atrial fibrillation and flutter | 3,134 (7.4) | 5,949 (8.3) | 0.034 | 3,409 (7.3) | 4,607 (9.2) | 0.070 | 2,363 (6.1) | 17,048 (16.0) | 0.318 | 1,559 (7.9) | 9,509 (7.2) | 0.025 |
| Allergic rhinitis | 2,965 (7.0) | 4,659 (6.5) | 0.018 | 3,129 (6.7) | 3,305 (6.6) | 0.003 | 2,712 (7.0) | 6,286 (5.9) | 0.047 | 1,342 (6.8) | 8,585 (6.5) | 0.013 |
| Urticaria | 551 (1.3) | 501 (0.7) | 0.055 | 607 (1.3) | 400 (0.8) | 0.044 | 503 (1.3) | 852 (0.8) | 0.051 | 236 (1.2) | 1,320 (1.0) | 0.015 |
| Food allergy status | 466 (1.1) | 788 (1.1) | 0.003 | 467 (1.0) | 500 (1.0) | 0.004 | 426 (1.1) | 1,589 (1.5) | 0.029 | 217 (1.1) | 1,584 (1.2) | 0.005 |
| Chronic allergic conjunctivitis | 169 (0.4) | 143 (0.2) | 0.027 | 187 (0.4) | 152 (0.3) | 0.014 | 155 (0.4) | 213 (0.2) | 0.025 | 79 (0.4) | 396 (0.3) | 0.015 |
| Gastro-esophageal reflux disease | 14,357 (33.9) | 20,931 (29.2) | 0.102 | 15,506 (33.2) | 15,375 (30.7) | 0.054 | 13,015 (33.6) | 35,587 (33.4) | 0.005 | 6,338 (32.1) | 41,077 (31.1) | 0.022 |
| Depressive episode | 8,851 (20.9) | 12,544 (17.5) | 0.087 | 9,761 (20.9) | 9,016 (18.0) | 0.074 | 8,018 (20.7) | 20,031 (18.8) | 0.046 | 3,770 (19.1) | 25,887 (19.6) | 0.014 |
| Nicotine dependence | 4,108 (9.7) | 8,960 (12.5) | 0.091 | 4,530 (9.7) | 5,809 (11.6) | 0.063 | 3,641 (9.4) | 14,278 (13.4) | 0.125 | 1,954 (9.9) | 15,057 (11.4) | 0.046 |
| Alcohol related disorders | 723 (1.7) | 1,577 (2.2) | 0.035 | 794 (1.7) | 951 (1.9) | 0.016 | 659 (1.7) | 2,877 (2.7) | 0.071 | 394 (2.0) | 2,377 (1.8) | 0.019 |
| Hypothyroidism | 7,496 (17.7) | 10,250 (14.3) | 0.094 | 8,080 (17.3) | 7,762 (15.5) | 0.049 | 6,818 (17.6) | 16,728 (15.7) | 0.050 | 3,336 (16.9) | 20,340 (15.4) | 0.041 |
| Hyperthyroidism | 551 (1.3) | 788 (1.1) | 0.021 | 607 (1.3) | 600 (1.2) | 0.012 | 504 (1.3) | 1,492 (1.4) | 0.002 | 236 (1.2) | 1,584 (1.2) | 0.003 |
| Type 2 diabetes complications |  |  |  |  |  |  |  |  |  |  |  |  |
| Kidney complications | 4,574 (10.8) | 8,171 (11.4) | 0.020 | 5,184 (11.1) | 7,562 (15.1) | 0.119 | 3,564 (9.2) | 22,375 (21.0) | 0.333 | 2,033 (10.3) | 16,642 (12.6) | 0.073 |
| Neurological complications | 5,040 (11.9) | 8,817 (12.3) | 0.012 | 5,791 (12.4) | 6,911 (13.8) | 0.044 | 4,222 (10.9) | 18,540 (17.4) | 0.189 | 2,052 (10.4) | 18,227 (13.8) | 0.105 |
| Circulation complications | 2,456 (5.8) | 3,727 (5.2) | 0.025 | 2,569 (5.5) | 2,904 (5.8) | 0.012 | 2,169 (5.6) | 9,376 (8.8) | 0.125 | 1,125 (5.7) | 7,660 (5.8) | 0.004 |
| Ophthalmic complications | 1,609 (3.8) | 2,293 (3.2) | 0.036 | 1,915 (4.1) | 2,253 (4.5) | 0.018 | 1,317 (3.4) | 5,967 (5.6) | 0.105 | 631 (3.2) | 5,811 (4.4) | 0.064 |
| Medication, n (%) |  |  |  |  |  |  |  |  |  |  |  |  |
| Lipid modifying agents | 19,651 (46.4) | 31,684 (44.2) | 0.045 | 21,811 (46.7) | 25,191 (50.3) | 0.073 | 16,928 (43.7) | 60,733 (57.0) | 0.268 | 8,409 (42.6) | 63,134 (47.8) | 0.104 |
| Antihypertensives | 3,812 (9.0) | 7,598 (10.6) | 0.055 | 3,877 (8.3) | 5,358 (10.7) | 0.083 | 3,370 (8.7) | 14,597 (13.7) | 0.159 | 1,816 (9.2) | 12,019 (9.1) | 0.003 |
| Inhaled corticosteroid | 18,719 (44.2) | 26,523 (37.0) | 0.147 | 20,410 (43.7) | 20,083 (40.1) | 0.073 | 16,773 (43.3) | 45,603 (42.8) | 0.011 | 8,113 (41.1) | 55,870 (42.3) | 0.026 |
| Systemic corticosteroid | 8,978 (21.2) | 14,121 (19.7) | 0.036 | 9,481 (20.3) | 9,515 (19.0) | 0.033 | 8,057 (20.8) | 22,056 (20.7) | 0.004 | 4,086 (20.7) | 25,359 (19.2) | 0.038 |
| Inhaled beta-2-adrenoreceptor agonists | 20,583 (48.6) | 34,766 (48.5) | 0.003 | 22,278 (47.7) | 24,690 (49.3) | 0.032 | 18,555 (47.9) | 55,938 (52.5) | 0.090 | 9,258 (46.9) | 64,719 (49.0) | 0.041 |
| Indacaterol | 0 (0) | 11 (0.0) | 0.018 | 0 (0) | 13 (0.0) | 0.022 | 0 (0) | 9 (0.0) | 0.015 | 0 (0) | 10 (0.0) | 0.013 |
| Olodaterol | 211 (0.5) | 358 (0.5) | 0.004 | 234 (0.5) | 300 (0.6) | 0.008 | 154 (0.4) | 1,173 (1.1) | 0.076 | 118 (0.6) | 660 (0.5) | 0.008 |
| Formoterol | 4,785 (11.3) | 6,309 (8.8) | 0.085 | 5,137 (11.0) | 4,959 (9.9) | 0.034 | 4,067 (10.5) | 12,679 (11.9) | 0.044 | 2,092 (10.6) | 13,472 (10.2) | 0.014 |
| Salmeterol | 3,472 (8.2) | 6,594 (9.2) | 0.035 | 3,689 (7.9) | 4,858 (9.7) | 0.062 | 3,060 (7.9) | 8,843 (8.3) | 0.015 | 1,519 (7.7) | 11,358 (8.6) | 0.033 |
| Leukotriene receptor antagonists | 6,056 (14.3) | 8,673 (12.1) | 0.066 | 6,258 (13.4) | 6,661 (13.3) | 0.004 | 5,384 (13.9) | 15,130 (14.2) | 0.008 | 2,546 (12.9) | 18,227 (13.8) | 0.027 |
| Tiotropium | 1,143 (2.7) | 2,509 (3.5) | 0.043 | 1,214 (2.6) | 1,903 (3.8) | 0.068 | 968 (2.5) | 5,221 (4.9) | 0.128 | 513 (2.6) | 4,622 (3.5) | 0.054 |
| Umeclidinium | 1,906 (4.5) | 1,505 (2.1) | 0.137 | 2,102 (4.5) | 1,252 (2.5) | 0.107 | 1,549 (4.0) | 5,226 (4.9) | 0.040 | 848 (4.3) | 4,358 (3.3) | 0.053 |
| Xanthines | 254 (0.6) | 645 (0.9) | 0.045 | 280 (0.6) | 550 (1.1) | 0.051 | 194 (0.5) | 1,385 (1.3) | 0.085 | 98 (0.5) | 1,056 (0.8) | 0.035 |
| Proton pump inhibitors | 13,171 (31.1) | 19,713 (27.5) | 0.081 | 14,712 (31.5) | 16,176 (32.3) | 0.017 | 11,737 (30.3) | 35,907 (33.7) | 0.071 | 5,626 (28.5) | 39,360 (29.8) | 0.029 |
| Biguanides | 14,230 (33.6) | 29,892 (41.7) | 0.169 | 16,487 (35.3) | 21,736 (43.4) | 0.165 | 12,705 (32.8) | 40,808 (38.3) | 0.115 | 6,494 (32.9) | 57,058 (43.2) | 0.213 |
| Thiazolidinediones | 551 (1.3) | 1,577 (2.2) | 0.070 | 794 (1.7) | 1,452 (2.9) | 0.083 | 542 (1.4) | 2,557 (2.4) | 0.075 | 315 (1.6) | 3,302 (2.5) | 0.061 |
| Alpha glucosidase inhibitors | 42 (0.1) | 72 (0.1) | <0.001 | 47 (0.1) | 100 (0.2) | 0.011 | 39 (0.1) | 213 (0.2) | 0.002 | 19 (0.1) | 264 (0.2) | 0.010 |
| Insulins and analogues | 9,148 (21.6) | 20,932 (29.2) | 0.176 | 10,742 (23.0) | 15,776 (31.5) | 0.193 | 7,631 (19.7) | 39,530 (37.1) | 0.393 | 3,790 (19.2) | 37,378 (28.3) | 0.215 |
| Sulfonylureas | - | - | - | 3,176 (6.8) | 10,267 (20.5) | 0.407 | 2,557 (6.6) | 16,409 (15.4) | 0.279 | 1,500 (7.6) | 20,208 (15.3) | 0.244 |
| Dipeptidyl peptidase 4 inhibitors | 1,228 (2.9) | 6,738 (9.4) | 0.275 | - | - | - | 1,123 (2.9) | 9,909 (9.3) | 0.271 | 947 (4.8) | 12,811 (9.7) | 0.187 |
| Sodium-glucose co-transporter 2 inhibitors | 6,395 (15.1) | 6,452 (9.0) | 0.188 | 7,566 (16.2) | 5,308 (10.6) | 0.166 | - | - | - | 2,605 (13.2) | 18,491 (14.0) | 0.022 |
| Glucagon-like peptide-1 analogues | 18,846 (44.5) | 6,595 (9.2) | 0.869 | 21,204 (45.4) | 4,357 (8.7) | 0.905 | 16,850 (43.5) | 19,925 (18.7) | 0.557 | - | - | - |
| Lab data |  |  |  |  |  |  |  |  |  |  |  |  |
| Eosinophil in blood, n (%)* | 34,304 (81.0) | 54,337 (75.8) |  | 36,944 (79.1) | 38,712 (77.3) |  | 30,601 (79.0) | 98,025 (92.0) |  | 15,928 (80.7) | 101,262 (76.6) |  |
| ≤ 150/uL | 13,171 (31.1) | 22,294 (31.1) | 0.001 | 14,105 (30.2) | 15,525 (31.0) | 0.019 | 11,776 (30.4) | 39,636 (37.2) | 0.143 | 6,079 (30.8) | 39,360 (29.8) | 0.021 |
| 150-300/uL | 12,366 (29.2) | 17,921 (25.0) | 0.093 | 13,218 (28.3) | 13,021 (26.0) | 0.052 | 10,962 (28.3) | 33,563 (31.5) | 0.069 | 5,704 (28.9) | 35,793 (27.1) | 0.040 |
| ≥ 300/uL | 8,767 (20.7) | 14,122 (19.7) | 0.026 | 9,621 (20.6) | 10,166 (20.3) | 0.007 | 7,863 (20.3) | 24,826 (23.3) | 0.074 | 4,145 (21.0) | 26,109 (19.7) | 0.033 |
| IgE in blood, n (%)* | 625 (1.5) | 499 (0.7) |  | 676 (1.4) | 388 (0.8) |  | 538 (1.4) | 981 (0.9) |  | 297 (1.5) | 1,263 (1.0) |  |
| ≥ 100 IU/ml | 212 (0.5) | 287 (0.4) | 0.026 | 280 (0.6) | 200 (0.4) | 0.027 | 194 (0.5) | 426 (0.4) | 0.011 | 97 (0.5) | 660 (0.5) | 0.007 |

TZP, Tirzepatide; SU, sulfonylurea; GLP-1RA, glucagon-like peptide-1 receptor agonist; DPP4i, dipeptidyl peptidase 4 inhibitor; SGLT2i, sodium-glucose cotransporter 2 inhibitor; SMD, standardized mean difference

*Number of patients with available data

**Supplemental Table 5**. Sensitivity analysis using a composite outcome of acute asthma exacerbation or all-cause mortality in tirzepatide versus comparator cohorts.

| Comparator | HR (95% CI) | *P* value | E-value  (95% LCL) |
| --- | --- | --- | --- |
| Tirzepatide vs. SU | 0.70 (0.63,0.77) | <0.001 | 2.11 (1.92) |
| Tirzepatide vs. DPP4i | 0.62 (0.55,0.70) | <0.001 | 2.61 (2.21) |
| Tirzepatide vs. SGLT2i | 0.84 (0.76,0.93) | <0.001 | 1.67 (1.36) |
| Tirzepatide vs. GLP-1RA | 1.02 (0.90,1.15) | 0.771 | 1.16 (1.00) |

CI, confidence interval; DPP4i, dipeptidyl peptidase 4 inhibitor; GLP-1RA, glucagon-like peptide-1 receptor agonist; HR, hazard ratio; LCL, lower confidence limit; SU, sulfonylurea; SGLT2i, sodium-glucose cotransporter 2 inhibitor

Supplemental Table 6. Missing data for continuous clinical variables before and after propensity score matching.

| Variables | TZP | SU | TZP | DPP4i | TZP | SGLT2i | TZP | GLP-1RA |
| --- | --- | --- | --- | --- | --- | --- | --- | --- |
| Before match |  |  |  |  |  |  |  |  |
| Sample size in each cohort | n = 42,351 | n = 71,684 | n = 46,705 | n = 50,083 | n = 38,736 | n = 106,549 | n = 19,740 | n = 132,081 |
| BMI | 10,053 (23.7%) | 30,681 (42.8%) | 12,003 (25.7%) | 22,788 (45.5%) | 9,025 (23.3%) | 42,193 (39.6%) | 5,508 (27.9%) | 41,076 (31.1%) |
| HbA1c | 28,545 (67.4%) | 32,832 (45.8%) | 30,218 (64.7%) | 22,688 (45.3%) | 26,457 (68.3%) | 51,889 (48.7%) | 12,871 (65.2%) | 66,305 (50.2%) |
| Eosinophil | 8,047 (19.0%) | 17,347 (24.2%) | 9,761 (20.9%) | 11,371 (22.7%) | 8,135 (21.0%) | 8,524 (8.0%) | 3,812 (19.3%) | 30,819 (23.3%) |
| IgE | 41,726 (98.5%) | 71,185 (99.3%) | 46,029 (98.6%) | 49,695 (99.2%) | 38,198 (98.6%) | 105,568 (99.1%) | 19,443 (98.5%) | 130,818 (99.0%) |
| After match |  |  |  |  |  |  |  |  |
| Sample size in each cohort | n = 25,712 | n = 25,712 | n = 23,013 | n = 23,013 | n = 30,292 | n = 30,292 | n = 19,740 | n = 19,740 |
| BMI | 7,997 (31.1%) | 7,843 (30.5%) | 8,285 (36.0%) | 8,124 (35.3%) | 8,573 (28.3%) | 8,331 (27.5%) | 5,508 (27.9%) | 5,468 (27.7%) |
| HbA1c | 15,068 (58.6%) | 14,913 (58.0%) | 12,750 (55.4%) | 12,888 (56.0%) | 18,267 (60.3%) | 17,812 (58.8%) | 12,871 (65.2%) | 12,910 (65.4%) |
| Eosinophil | 6,325 (24.6%) | 5,967 (23.2%) | 5,732 (24.9%) | 5,660 (24.6%) | 6,787 (22.4%) | 6,482 (21.4%) | 3,812 (19.3%) | 3,807 (19.3%) |
| IgE | 25,414 (98.8%) | 25,465 (99.0%) | 22,748 (98.8%) | 22,772 (99.0%) | 29,934 (98.8%) | 29,929 (98.8%) | 19,443 (98.5%) | 19,450 (98.5%) |

Values are presented as n (%) missing for each continuous variable.

Supplemental Figure 1. Stratified analysis of the risk of the primary outcome comparing tirzepatide (TZP) with sulfonylureas

BMI, body mass index; CVD, cardiovascular disease; HF, heart failure; COPD, chronic obstructive pulmonary disease; eGFR, estimated glomerular filtration rate; HbA1c, hemoglobin A1c; ICS, inhaled corticosteroid

Supplemental Figure 2. Stratified analysis of the risk of the primary outcome comparing tirzepatide (TZP) with dipeptidyl peptidase-4 inhibitors (DPP-4i) Sulfonylureas

BMI, body mass index; CVD, cardiovascular disease; HF, heart failure; COPD, chronic obstructive pulmonary disease; eGFR, estimated glomerular filtration rate; HbA1c, hemoglobin A1c; ICS, inhaled corticosteroid

Supplemental Figure 3. Stratified analysis of the risk of the primary outcome comparing tirzepatide (TZP) with sodium-glucose cotransporter 2 inhibitors

BMI, body mass index; CVD, cardiovascular disease; HF, heart failure; COPD, chronic obstructive pulmonary disease; eGFR, estimated glomerular filtration rate; HbA1c, hemoglobin A1c; ICS, inhaled corticosteroid

Supplemental Figure 4. Stratified analysis of the risk of the primary outcome comparing tirzepatide (TZP) with glucagon-like peptide-1 receptor agonists

BMI, body mass index; CVD, cardiovascular disease; HF, heart failure; COPD, chronic obstructive pulmonary disease; eGFR, estimated glomerular filtration rate; HbA1c, hemoglobin A1c; ICS, inhaled corticosteroid
